# Characterization of the Conformational Fluctuations in the Josephin Domain of Ataxin-3

**DOI:** 10.1016/j.bpj.2014.10.008

**Published:** 2014-12-16

**Authors:** Domenico Sanfelice, Alfonso De Simone, Andrea Cavalli, Serena Faggiano, Michele Vendruscolo, Annalisa Pastore

**Affiliations:** 1 MRC National Institute for Medical Research, London, United Kingdom; 2 Division of Molecular Biosciences, Imperial College London, London, United Kingdom; 3 Institute for Research in Biomedicine, 6500 Bellinzona, Switzerland; 4 Department of Chemistry, University of Cambridge, Cambridge, United Kingdom; 5 Department of Basic and Clinical Neurosciences, King’s College London, London, United Kingdom

## Abstract

As for a variety of other molecular recognition processes, conformational fluctuations play an important role in the cleavage of polyubiquitin chains by the Josephin domain of ataxin-3. The interaction between Josephin and ubiquitin appears to be mediated by the motions of *α*-helical hairpin that is unusual among deubiquitinating enzymes. Here, we characterized the conformational fluctuations of the helical hairpin by incorporating NMR measurements as replica-averaged restraints in molecular dynamics simulations, and by validating the results by small-angle x-ray scattering measurements. This approach allowed us to define the extent of the helical hairpin motions and suggest a role of such motions in the recognition of ubiquitin.

## Introduction

Increasing evidence indicates that conformational fluctuations play an important role in molecular recognition (1, 2, 3, 4, 5, 6, 7, 8, 9). It is therefore of primary importance to develop methods to correlate the dynamics of proteins with the mechanisms by which they recognize their molecular partners (10, 11, 12, 13, 14, 15). An approach recently described to characterize the dynamics of proteins is based on the use of chemical shifts (CSs) and residual dipolar couplings (RDCs) obtained from NMR measurements as replica-averaged structural restraints in molecular dynamics (MD) simulations (16, 17, 18, 19). This approach allows the study of conformational fluctuations occurring up to the millisecond timescale. Application of the method led to the characterization of the collective motions in ribonuclease A, lysozyme, and calmodulin (16, 17, 18). In this work, we extend the applicability of this method to the larger and more complex structure of the Josephin domain. This is an interesting target both in view of its biological role and its implication that disease:ataxin-3 is the protein responsible for the inherited neurodegenerative Joseph-Machado disease (also called spinocerebellar ataxia type-3) (20, 21, 22, 23) and a member of the polyglutamine disease family (24).

Ataxin-3 is a deubiquitinating enzyme that binds and cleaves polyubiquitin chains from proteins targeted for proteasomal degradation (20, 25, 26, 27) with a preference for linear substrates (26, 28, 29) and K48/K63-linked polyubiquitin chains (30, 31). The proteolytic activity of ataxin-3 is intrinsically low but is significantly increased by self-ubiquitination that occurs specifically at K117 (31, 32).

Ataxin-3 consists of an N-terminal Josephin domain, where the deubiquitinating enzymatic activity resides, and an unstructured C-terminus, which contains the polyglutamine tract and multiple copies of the ubiquitin interacting motif. The structures of the free and the ubiquitin-bound Josephin domain have been determined (29, 33, 34, 35). Josephin contains two subdomains, a globular one containing the catalytic active site and *α*-helical hairpin formed by *α*-helices 2 and 3. Open and closed conformations of Josephin have been described, which differ for the relative orientation of the two subdomains (33, 35). Previous measurements have suggested that the helical hairpin is not a rigid structural element but rather has dynamical properties quite different from those of the rest of the domain (33, 34). The presence of the hairpin is rather unusual in the cysteine protease fold family, suggesting a role in substrate or partner specificity recognition for this secondary structure element (36). Josephin contains two highly conserved ubiquitin binding sites that are positioned on either side of the helical hairpin (34). Site 1 is essential for enzymatic activity (37), whereas site 2 is thought to confer ubiquitin-chain linkage preference and overlaps with the interaction surface of the ubiquitin-like domain of hHR23B (33, 37). Interestingly, the dynamics of the hairpin are significantly dumped upon interaction of ubiquitin in site 1 (33, 34). Understanding the extent and nature of the helical hairpin dynamics is thus important as these factors may play a predominant role in the enzymatic function of ataxin-3.

Here, we characterized the dynamics of Josephin by determining an ensemble of conformations representing the conformational fluctuations of this protein domain. Our results, which are experimentally validated by small-angle x-ray scattering (SAXS) measurements, reveal the presence of concerted motions that affect the mutual orientation of the globular and the *α*-helical hairpin subdomains. Comparison of the free energy landscape of the unbound state of Josephin and the structure of this domain in a ubiquitin-bound state illustrates the role of conformational fluctuations in the molecular recognition process.

## Materials and Methods

### Protein production

The Josephin domain (residues 1–182 of ataxin-3) was produced as previously described as a glutathione S-transferase (GST) fusion protein with a cleavage site for the recombinant Tobacco Etch Virus (rTEV) protease (38). In short, the protein domain was overexpressed in *Escherichia coli* strain BL21(DE3) cells at 37°C and purified using a Glutathione Sepharose affinity matrix (Amersham Biosciences, Little Chalfont, UK). The Josephin domain was cleaved from glutathione S-transferase using a hexahistidine-tagged rTEV protease and separated from rTEV protease using Ni-NTA agarose (QIAGEN. Crowly, UK) leaving two nonnative residues (GA) at the N-terminus of the protein. Isotopically ^15^N-labeled and ^13^C/^15^N double-labeled samples were overexpressed in *E. coli* by growing it on minimal media containing 1 gl^-1 15^N-ammonium sulfate and 2 gl^-1 13^C-glucose and purified as described.

### NMR measurements

A series of NMR experiments were recorded at 298 K and 303 K (bicelles) on Bruker Avance spectrometers operating at 600 and 700 MHz proton frequencies.^15^N–^1^H RDCs were measured as the difference in splitting between isotropic (no alignment, *J*) and anisotropic (aligned, *J*+ RDC) samples from two-dimensional heteronuclear single quantum correlation-based experiments (39). 2048 complex points were acquired in the direct ^1^H dimension and 200 increments in the indirect ^15^N dimension with spectral widths of 10,000 and 1400 Hz for ^1^H and ^15^N, respectively. All data were processed in NMRPipe (40) and analyzed by NMRview (41). Virtually complete assignment of the spectrum has been previously described (33, 34, 42).

### RDC restraints

RDCs were measured in three different aligning media: stretched polyacrylamide gels, doped bicelles, and *Pf1* phages. 5% polyacrylamide gels were polymerized in polycarbonate tubes (5.5 mm inner diameter) from a mixture of acrylamide, bis-acrylamide in a final 20 mM NaH_2_PO_4_/Na_2_HPO_4_buffer (pH 6.5) using ammonium persulfate, as precursor and tetramethylethylenediamine as catalyst. The gels were cut into 12 mm pieces and washed in milliQ water (2000:1) for 2 days. Subsequently, the gels were dehydrated at 37°C for 24 h. A dried polyacrylamide gel was rehydrated with a protein sample for 16 h at 37°C. A gel stretching apparatus (New Era Enterprises) (43) was used to transfer the gel into an open-ended NMR tube, leading the gel to stretch to ∼1.6 times its original length. Lipid bicelles were prepared from powdered lipids, obtained from Avanti Polar Lipids, and 50 mg dihexanoyl-phosphatidylcholine was combined with 200 mg 1,2-Dimyristoyl-*sn*-glycero-3-phosphocholine (q = 2.6). Sodium cholesteryl sulfate (Sigma) was added to 13.4% with respect to 1,2-Dimyristoyl-*sn*-glycero-3-phosphocholine moles. Lipids were diluted to 20% w/v lipids with 90% H_2_O and 10% D_2_O phosphate buffer (20 mM at pH 6.8). Filamentous phages were purchased from ASLA-Biotech at a ∼50 mg/ml concentration. Samples aligned in *Pf1* were obtained by pipetting the phages in the protein solution to a final 20 mg/ml, with and without NaCl (500 mM). A total of 115 RDCs measured in *Pf1* at high salt concentration were used for MD simulations because of producing higher quality spectra. The RDC data obtained for weakly aligned bicelles, polyacrylamide gels, and low and high salt phages are reported in Tables S1–S4 in the Supporting Material. RDC restraints were calculated by adopting a structure-based method of the alignment tensor that accounted for the interaction between the protein and the alignment media (44). We employed the RDC data recorded in *Pf1*, with a concentration of 150 mM NaCl.

In this approach, the RDC restraints are imposed by adding a pseudoenergy term (*E*^*RDC*^) to a standard molecular mechanics force field (*E*^*FF*^) to obtain a hybrid experimental-empirical force field (*E*^*Total*^) for MD simulations

(1) $$E^{Total}=E^{FF}+E^{RDC},$$

where the pseudoenergy term is given by

(2) $$E^{RDC}=\alpha {\left(\text{RD}{\text{C}}^{\text{calc}}-\text{RD}{\text{C}}^{\exp}\right)}^{2},$$

*α* is the restraint force constant, and RDC^exp^ and RDC^calc^ are the experimental and calculated residual dipolar couplings, respectively (16, 45). The force constants for CS and RDC restraints were calibrated separately. Usually this procedure results in stable simulations. In some cases, it is possible to observe inconsistencies between the two sets of restraints. This is an indication of considerable experimental errors. We were careful to check that this is not the case for Josephin.

The restraints were imposed as averages over 16 replicas of the system (45) by computing independently the alignment tensor of each replica (45). RDC scaling due to orientational averaging was implemented in the method automatically. Each structure-based alignment medium contains an order parameter that effectively accounts for the orientational averaging, as previously described (44).

The alignment tensors were computed every 250 steps by following a structure-based protocol (45). An initial equilibration simulation at 300 K was run, during which the agreement between calculated and experimental data was allowed to converge by gradually raising the restraint force constant from zero to a value set on the basis of the level of agreement with experimental data. This was done to provide the best agreement between calculated and experimental data. In this study the optimal force was determined to be 1000 J/(mol Hz^2^). In the annealing cycles, when the system samples were at high temperatures, the force constant was scaled down to 10% of its maximum value.

Subsequently, a series of 50 cycles of simulated annealing between 300 K and 400 K were carried out to sample effectively the conformational space. Each cycle was carried out for 500 ps in each replica (total sampling of 8 ns) using an integration step of 1 fs. The final 100 ps of each cycle, which were equilibrated at 300 K, were used to compose the equilibrated ensemble, totaling 9600 structures, by collecting structures from each replica in intervals of 5 ps. The convergence of the calculations was checked by monitoring the time evolution of the radius of gyration, secondary structure content and exploration of the secondary structure populations, and by analyzing the agreement with experimental Nuclear Overhauser Effect (NOE) data. It is worth noting that the timescales sampled by RDC differ from those associated with NOEs. Nevertheless, the use of chemical shifts in our simulations justifies the use of NOEs for validation as these two observables sample similar timescales. The initial 20 cycles were not considered in the analyses. Experimental errors in the employed RDC are within the tolerance of the restrained simulation method (46).

### CS restraints

In addition to RDC restraints, we also used CS restraints, which were imposed using the CamShift method (47) using the CSs of the HN, H*α*, C*α*, C*β*, CO, and N atoms. We followed a MUMO scheme (48) in which the RDC restraints are imposed over the 16 replicas, whereas the CS restraints are imposed over rotating groups of four replicas (49). This choice was made to reduce over fitting associated to CS restraints (49), whereas for RDC the optimal number of replicas was previously determined to be 16 (46).

### MD simulations

The simulations were carried out using a modified version of the Gromacs package (50) that implements RDCs and CSs as structural restraints by using the Amber99SB force field (51) with improved parameters for backbone and side-chain atoms (52). The initial coordinates (Protein Data Bank (PDB) code 1yzb) were inserted in a rectangular box (starting dimensions 86.6 × 57.1 × 56.7 Å^3^) and solvated by 8363 explicit TIP3P water molecules (53). Bonds were constrained by the LINCS algorithm (54). The particle-mesh Ewald method (55) was used to account for the electrostatic contribution to nonbonded interactions (grid spacing of 0.12 nm). Arg and Lys residues were assumed to be positively charged, Asp and Glu residues were negatively charged, and histidines were assumed to be neutral. The net charge of the system was neutralized by the addition of Cl^−^ and Na^+^ ions. The system was equilibrated with external temperature and pressure baths (NPT ensemble) by using the v-rescale (56) and Berendsen (57) algorithms and coupling time steps respectively of 0.1 ps and 1.0 ps.

### SAXS measurements

SAXS data were collected on the EMBL P12 beamline at the PETRA III storage ring, DESY, Hamburg. Experiments were performed at Josephin concentrations of 1.1, 2.1, and 4.3 mg/mL using PILATUS 2 M pixel detector (DECTRIS, Baden, Switzerland), sample-detector distance 3.1 m, wavelength *λ* = 1.25 Å, covering the momentum transfer range 0.003 < *s* < 0.45 Å^−1^ (*s* = 4*π*sin(*θ*)/*λ* where 2*θ* is the scattering angle). To check for radiation damage, 20 50-ms exposures were compared; no radiation damage effects were observed. The data were averaged after normalization to the intensity of the incident beam and the scattering of the buffer was subtracted. The difference data were extrapolated to zero solute concentration following standard procedures. All data manipulations were performed using the program package PRIMUS (58).The experimental scattering profiles were further compared to those back-calculated for the RDC-CS ensemble, for the two NOE ensembles of free Josephin (1zyb and 2aga) and for the crystallographic structure of a Josephin-ubiquitin complex (3o65) using the FoXS approach (59).

## Results

### NMR data collection

Several features make the task of characterizing the dynamics of the Josephin domain using replica-averaged MD simulations. This domain comprises 182 residues, thus being rather longer than the examples previously examined by MD simulations with replica-averaged RDC restraints (i.e., ribonuclease A and lysozyme) (45, 46). As a consequence, the NMR spectrum has appreciable overlap making it difficult to record a semicomplete set of RDCs. In addition, the media necessary for sample alignment in the magnetic field were limited by the high tendency of Josephin to aggregate in most environments (33, 60). To circumvent the latter problem we tested several alignment media, such as bicelles (61), acrylamide gels (62), and phages (63).

The data in bicelles, gels, and phages at low salt were however sparse and showed only weak alignment (see Tables S2–S4). In gel and in phages at low salt hardly any RDC is greater than 4 Hz. Bicelles were not significantly better. Some of the reasons for this behavior can be explained by considering that gels are confined environments, which greatly enhance the tendency of Josephin to aggregate as already previously reported (33). Bicelles do not always align sufficiently. The results could, in principle, be improved by increasing the temperature but this could not be done with Josephin, again because it would promote aggregation. The use of phages at low salt proved also not to be ideal because Josephin has a negative pI, as also phages do. After repetitively trying to use all the RDCs we decided to use only the data in phages at high salt. The final data were collected in 150 mM NaCl. We had found similar conditions to be optimal also for other proteins. A total of 115 RDCs measured in *Pf1* at high salt concentration were used for the calculations.

### Characteristics of the RDC-CS ensemble

**Figure 1:**
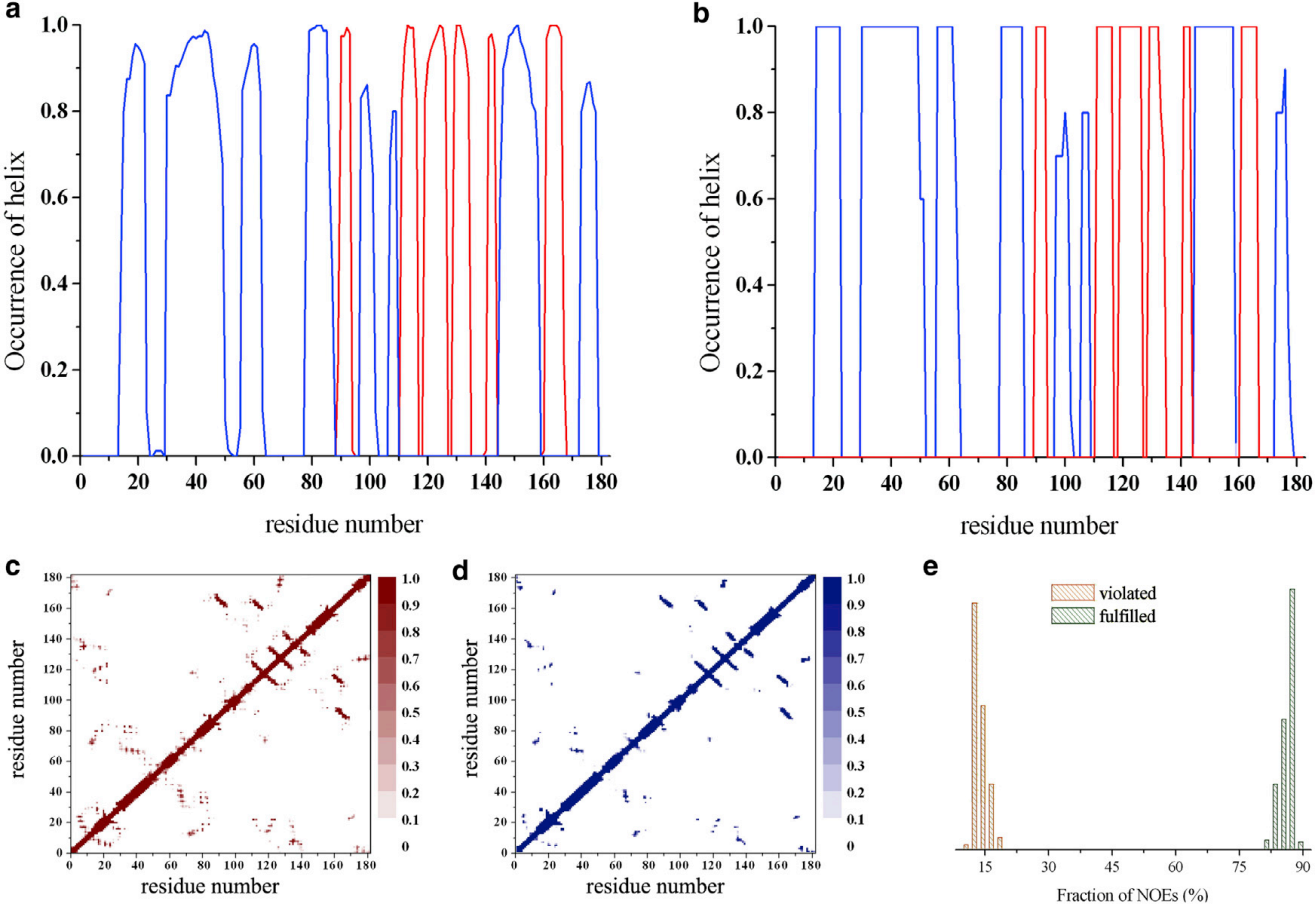
Structural comparison of the RDC-CS and NOE (1yzb) ensembles. (*a* and *b*) Occurrence of *α*-helical (*blue*) and *β*-sheet (*red*) structures along the sequence: RDC-CS ensemble (*a*), and NOE ensemble (*b*). In the helical populations we considered both *α*-helices and 3_10_-helices. (*c* and *d*) Contact maps of the RDC-CS ensemble (*c*) and NOE ensemble (*d*). The contact map reports the occurrence of residue-residue contacts in the ensemble. For each pair of residues a contact is counted if the C^*α*^-atoms are at a distance lower than 8 Å. The calculation is performed on individual structures of the ensemble. (*e*) Validation of the RDC-CS ensemble with NOE data. For each structure of the RDC-CS ensemble experimental NOEs are checked by using the distance limits as employed in the NOE structural refinement (PDB code 1yzb). The plot reports the distribution of percentage of NOEs fulfilled (*green*) and violated (*orange*) by individual structures of the RDC-CS ensemble. To see this figure in color, go online.

The structural ensemble based on RDC and CS restraints (RDC-CS ensemble) that we describe in this work is composed of 9600 conformations. We first checked that the new ensemble fulfills all the available NMR observables. Comparison of the RDC-CS ensemble with our previously determined NOE-based ensemble (1yzb) showed that the secondary structure contents according to the DSSP program (64) are similar (Fig. 1, *a* and *b*), with only a minor reduction of secondary structure populations in the RDC-CS ensemble, as expected from its increased dynamic content. Likewise, the contact maps are similar (Fig. 1, *c* and *d*), confirming that the tertiary structures are in close agreement. Correspondingly, the RDC-CS ensemble is also in good agreement with the experimental NOE data (Fig. 1 *e*).

### Validation of the RDC-CS ensemble by SAXS

**Figure 2:**
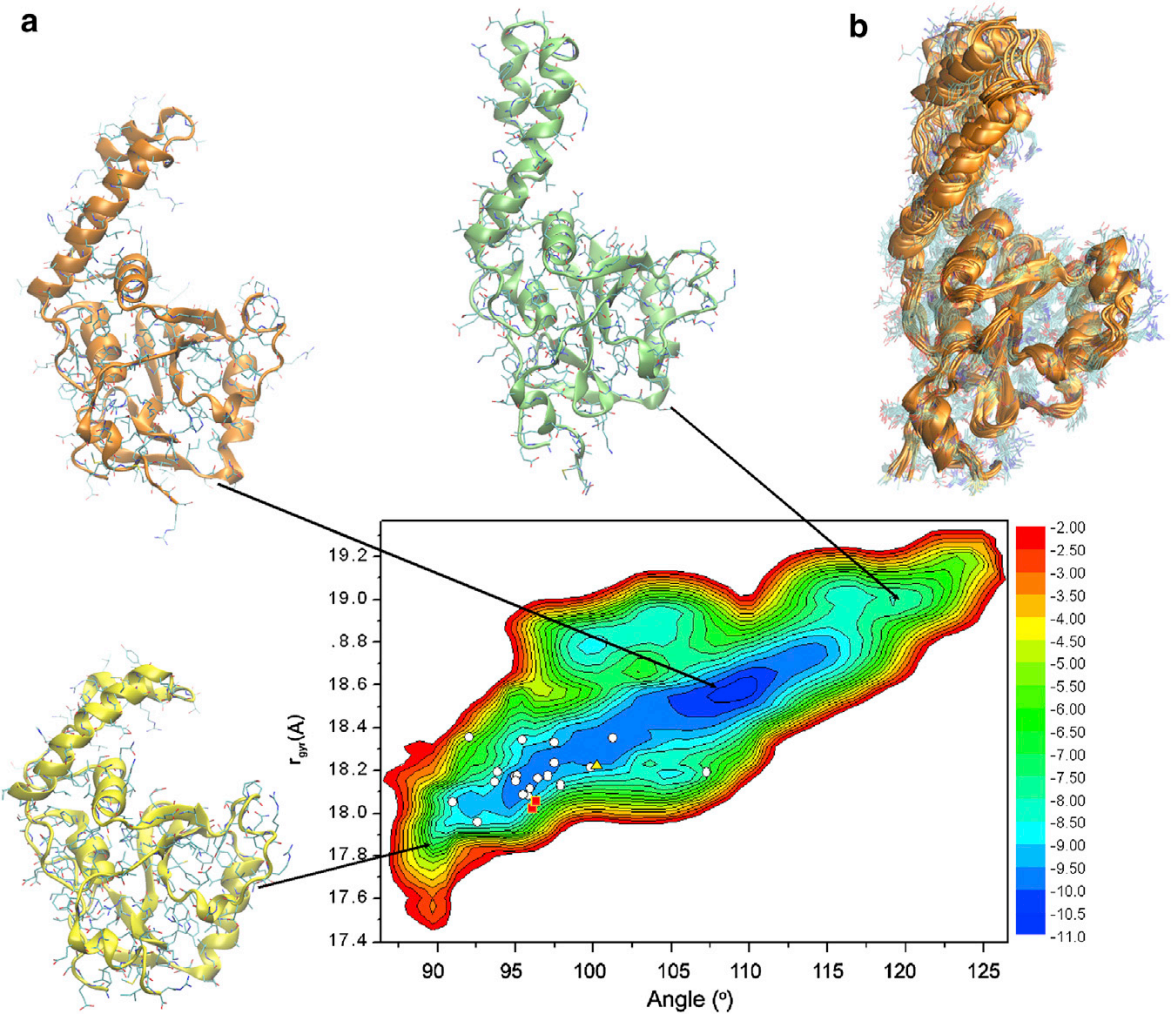
Small-angle x-ray scattering analysis of Josephin. (*Left*) Comparison of the experimental (*black line*) and calculated SAXS profiles for the RDC-CS ensemble (*red line*). (*Right*) For comparison the same plot is shown for the PDB structures 1yzb, 2aga, and 3o65. (*b*, *inset*) Alignment of the PDB structures 1yzb (*green*), 2aga (*cyan*), and 3o65 (*yellow*) to a representative structure of the RDC-CS ensemble (*magenta*). To see this figure in color, go online.

To validate the RDC-CS ensemble experimentally, we employed SAXS measurements. Fitting of the SAXS data was carried out using the FoXS software (59). This approach allows the analysis of a complete ensemble without preselection, thus permitting to retain the full richness of the conformational landscape provided by the replica-averaged MD simulations. We found that the RDC-CS structural ensemble is in excellent agreement with the SAXS data (Fig. 2 *A*).

We compared the agreement with available structures of Josephin. In addition to our original NOE-based ensemble (1yzb), there is another NOE-based NMR ensemble (2aga), which significantly differs for the orientation of the helical hairpin, which, in this structure, packs against the main domain. The only high-resolution structure (2.7 Å) available is the crystal of the ataxin-3 like protein ATXN3L in a complex with ubiquitin (3o65) (29). This homolog shares with Josephin 85% identity. In the complex, ubiquitin is covalently attached to the active site Cys-14 sites in site 1.

Both NOE-derived ensembles (1yzb and 2aga) are in poorer agreement than the RDC-CS structural ensemble (Fig. 2 *B*). The *χ*^2^ for the 1yzb and 2aga ensembles are respectively 1.94 and 2.38. The *χ*^2^ value for the x-ray structure 3o65 is 3.42. These numbers should be compared to 1.2 ± 0.1 for the RDS-CS ensemble. Interestingly, albeit different in the hairpin orientations, both ensembles score significantly better than the crystallographic structure of the complex indicating the presence of a genuine dynamic equilibrium in solution for the free protein. The better fit of the RDC-CS ensemble as compared to the NOE derived ensemble 1yzb indicates the necessity of accounting for larger levels of flexibility of the *α*-helical hairpin (Fig. 2 *b*, *inset*).

These results give us confidence about the validity of our approach to describe the molecular motions of the Josephin domain.

### Analysis of the conformational fluctuations of Josephin

**Figure 3:**
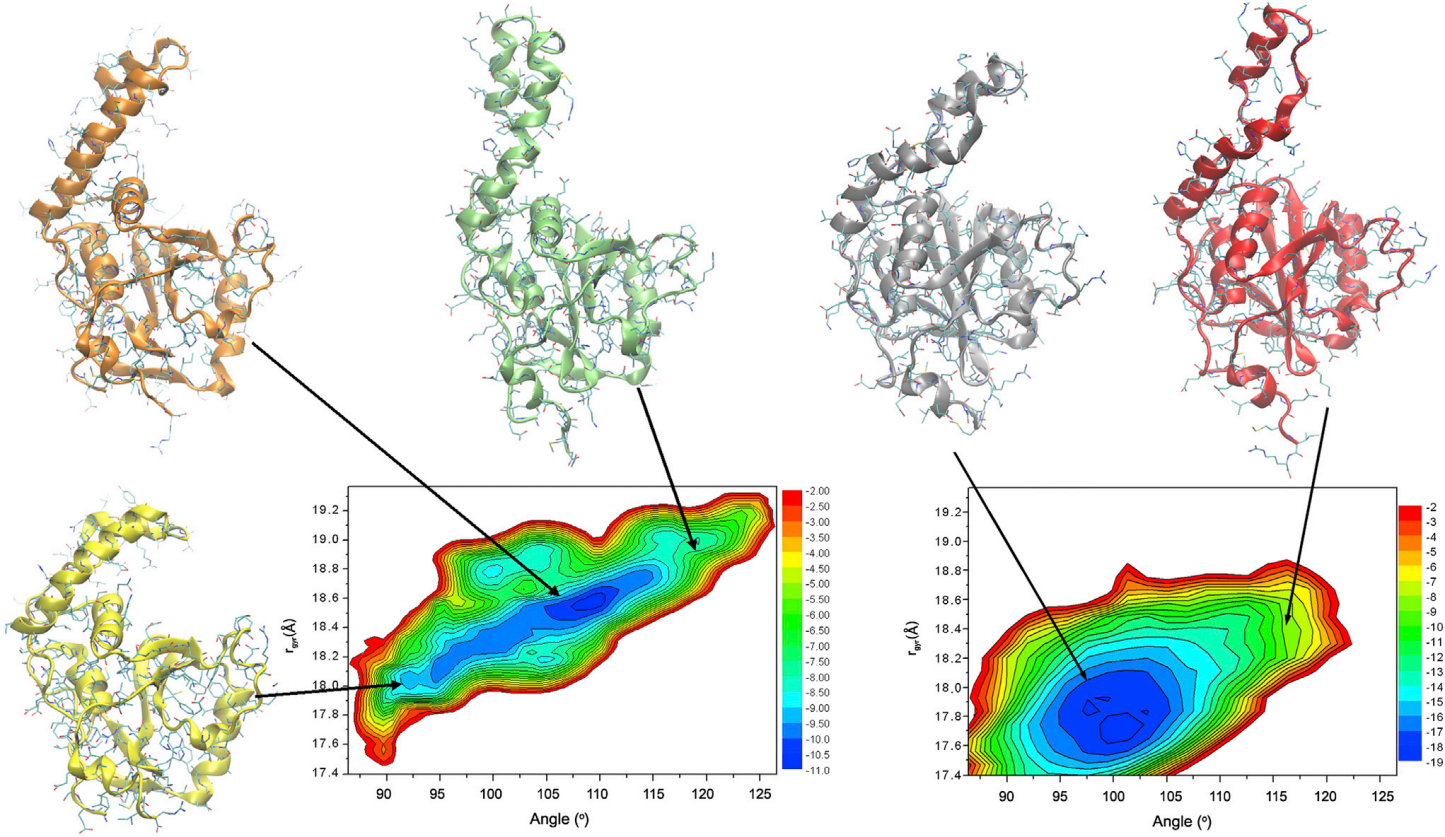
Free energy landscapes of Josephin and representative structures. The left panel corresponds to the restrained ensemble. The free energy landscape is projected along two reaction coordinates, the radius of gyration (*y* axis) and the bending angle (*x* axis). The energy values are in k_B_T units. Representative structures within the RDC-CS ensemble for Josephin conformations are also shown on the left panel at small (*yellow*), intermediate (*orange*), and large (*green*) values of the bending angle. The structures are superimposed by using the C^*α*^ atoms of the globular subdomain for least square fitting. Individual structures from the bound state are shown by white circles (PDB code 1yzb), red squares (PDB code 3o65), and black triangles (PDB code 2aga). These results illustrate how the compact conformations in the free state resemble those in the bound state. The right panel shows, as a control, the landscape for the unrestrained ensemble. Two representative structures are shown on the top in blue and red having angles of 98° and 115°, respectively. To see this figure in color, go online.

Having validated our results experimentally, we were in the position of using the new ensemble to characterize the conformational fluctuations of Josephin quantitatively. We projected the RDC-CS ensemble on two reaction coordinates accounting for local and global properties (Fig. 3, *left panel*).

As the first reaction coordinate, we selected a large-scale bending angle between the globular and the helical hairpin subdomains. The angle is computed from the centers of mass of the C^*α*^-atoms from three distinct protein regions: region I, which spans residues 111–113, 122–125, and 162–165 (globular subdomain), region II (hinge), which spans residues 32–35, and region 3 (loop), which spans residues 45–48 and 58–61. We used as the second reaction coordinate the radius of gyration, which is a global property influenced by the coordinates of all C^*α*^-atoms of the protein. The resulting free energy landscape features a main conformational basin describing large-scale motions giving rise to the large variability of the mutual orientation of the *α*-helical hairpin and the globular subdomains. These motions modulate significantly the structural properties of the protein by changing the shape and the characteristics of the protein surface. As a control, we plotted the distribution corresponding to a structural ensemble resulting from an unrestrained simulation, which is significantly different (Fig. 3, *left panel*). The sampling tends to close the hairpin slightly more and the minimum is shallower, clearly indicating the important contribution of the restraints. We compared and validated the restrained and unrestrained ensembles against the SAXS data. The *χ*^2^ for the restrained ensemble is 1.01 ± 0.02, whereas is 1.15 ± 0.13 for the unrestrained one. The close similarity of these numbers reflects, of course, the effect of the common force field but the slightly smaller value obtained for the restrained ensemble solely reflects the importance of the restraints.

### Comparison of the structures of Josephin in the free and ubiquitin-bound states

**Figure 4:**
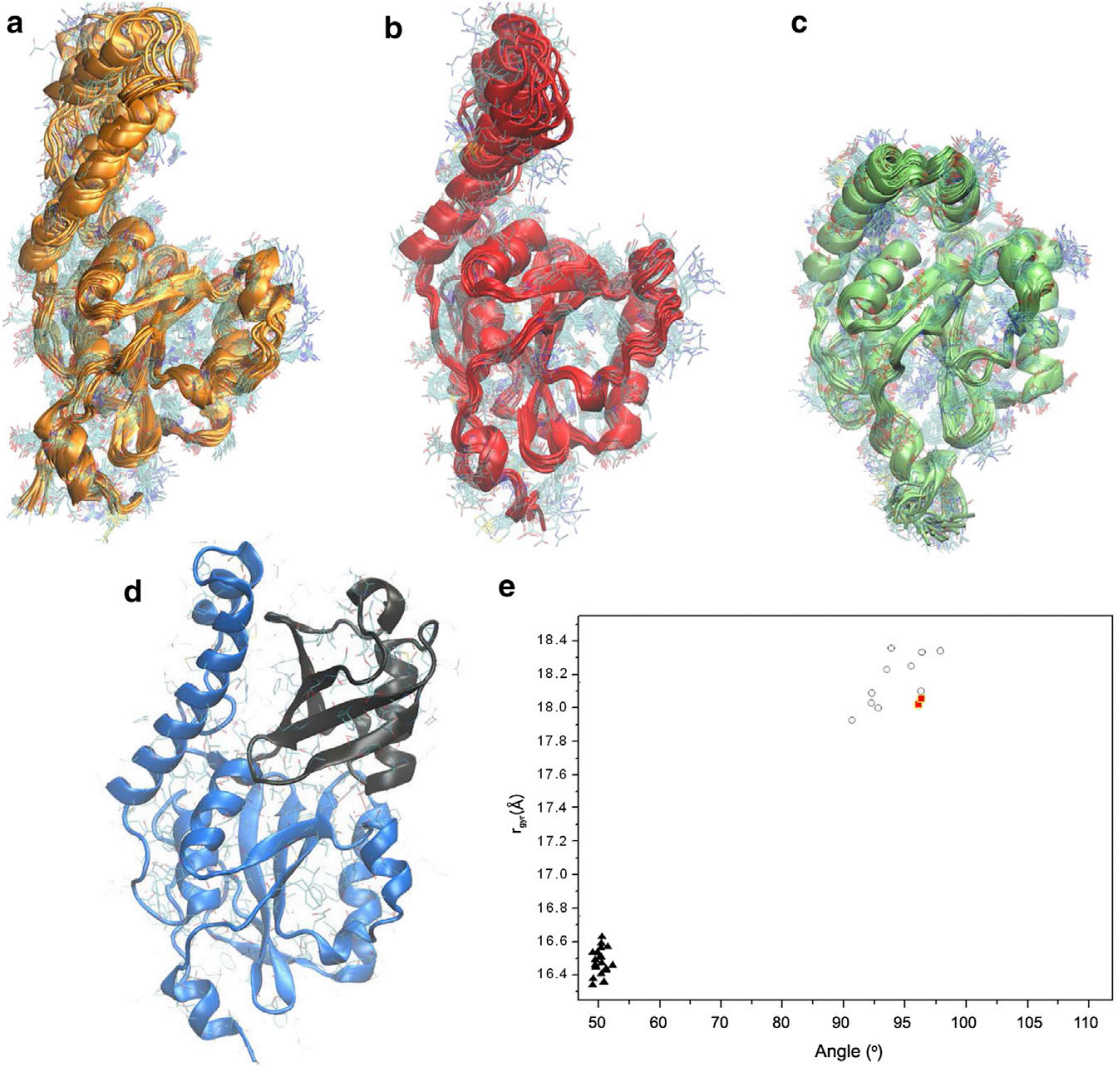
Comparison of bound and free structures. Bundles from the RDC-CS ensemble (*a*), the NOE ensemble 1yzb (*b*), the NOE ensemble 2aga (*c*) of Josephin in the free state are compared to the x-ray structure of ubiquitin-bound Josephin 3o65 (*d*). Panel *E*, values adopted along the coordinates as used in Fig. 3 calculated on the individual structures. These are shown by white circles (PDB code 1yzb), red squares (PDB code 3o65), and black triangles (PDB code 2aga). To see this figure in color, go online.

We compared the free energy landscape of the RDC-CS ensemble (Fig. 3) with the PDB structures of Josephin both in the free- and in the ubiquitin-bound states (Fig. 4). The NOE-derived 1yzb ensemble is more compact having a radius of gyration of 17.2 ± 0.1Å as compared to a value of 18.7 ± 0.3 Å for the RDC-CS ensemble. For comparison, the radius of gyration calculated for 2aga and 3o65 are respectively 16.7 ± 0.1Å and 19.8 ± 0.3 Å. The agreement of the radius calculated for the 2aga ensemble is thus significantly worse.

Projection of these structures onto the free-energy landscape produced for the RDC-CS ensemble is very informative. Beyond the overall similarity, the RDC-CS ensemble shows a much larger variability of the mutual orientation of the globular domain and the *α*-helical hairpin as compared to the NOE-based ensemble, which does not occupy the minimum of the free energy landscape but systematically clusters in the lower corner of the free-energy landscape, showing smaller bending angles and reduced values of the radius of gyration (Fig. 4 *e*). The 2aga ensemble is far away from the minimum confirming that this structure is not representative of the Josephin structure as previously suggested (42). The most interesting observation is, however, that the bending angle in the bound state of Josephin selects one of the more open conformations. This result indicates that binding affects both the preferential orientation of the hairpin and the fluctuations between the two subdomains blocking the hairpin in a preferred blocked conformation.

We can thus conclude that the methodology described here can be used both for structure validation and to describe the conformational fluctuations of proteins with a multidomain architecture.

## Discussion

Understanding how proteins recognize their functional partners has enormously improved in recent years. An important lesson from these studies is that, also when in their native states, proteins undergo structural fluctuations with timescales ranging from picoseconds to seconds and beyond. These motions are of direct biological relevance as they may influence a wide variety of processes, including enzymatic catalysis, ligand binding, and the formation of biomolecular complexes.

In this work, we have determined an ensemble of structures representative of the dynamics of the Josephin domain of ataxin-3 to investigate the mechanism of molecular recognition between this protein domain and ubiquitin. We applied a recently developed structure refinement approach that involves the incorporation of RDC and CS experimental data as replica-averaged structural restraints in MD simulations, and allows the description of motions in the nanosecond to millisecond timescale without the use of NMR relaxation data. This methodology has so far proven to be successful to study the dynamics of small globular proteins such as lysozyme and ribonuclease A (16, 45). As compared to these proteins, Josephin is a more challenging example because, in addition to being a 182-residue protein domain with a spectrum dominated by large resonance overlap, it has a strong tendency to aggregate in solution. This aspect prevents alignment strategies such as those based in confined media. At the same time, however, the dynamics of Josephin seems to play an important role in protein function as it takes part in substrate recognition and specificity (58).

The ensemble of structures representing the free state of Josephin determined in this work reveals the presence of motions of relatively large amplitude of the helical hairpin as compared to the rest of the structure while keeping its secondary structure conserved. We independently validated our results with SAXS data, which fully endorse the presence of large fluctuations of the hairpin. We have shown that our approach is also useful for a posteriori structure validation. The results that we have described provide, in fact, further support to previous evidence that excluded conformations of Josephin in which the hairpin is so close to the other subdomain to be packing against it (42). The hairpin flexibility is thus limited to motions that do not lead to the complete closing of the groove allowing ubiquitin to enter in its binding site.

Comparison of the free energy landscape of the unbound state of Josephin and the structure of this domain in a ubiquitin bound state demonstrate that, upon binding to ubiquitin, the conformational entropy of the hairpin decreases (33, 34). These conclusions, which emerge from the analysis of the structural ensembles, are complemented by those obtained by independent measurements of ^15^N relaxation of the free and bound states, which show that ubiquitin binding appreciably reduces the *α*-helical hairpin motions (34). A similar role of the conformational fluctuations has been observed for the protein kinase PKA-C (5) and for calmodulin (65) and seems to provide a specific advantage on molecular recognition.

We can thus conclude that the methodology described here can be used to study protein dynamics and motions that escape other descriptions. This approach may be of particular interest to assist in the difficult problem of defining conformational fluctuations and for obtaining a decomposition of the fast and slow timescale motions in complex systems.

## Supporting Material

Document S1. Four tables

Document S2. Article plus Supporting Material
