# Supplementary material for: Characterization of the Conformational Fluctuations in the Josephin Domain of Ataxin-3
Source: Biophys J. 2014 Dec 16;107(12):2932–40. doi: 10.1016/j.bpj.2014.10.008 (PMC4269769; doi:10.1016/j.bpj.2014.10.008)
Supplement: Document S1. Four tables [file mmc1.pdf]

The role of conformational fluctuations in the recognition of the Josephin domain of ataxin-3 with ubiquitin

Domenico Sanfelice[1‡], Alfonso De Simone[2], Andrea Cavalli[3,4], Serena Faggiano[1], Michele Vendruscolo[4], Annalisa Pastore[5*]

[1]*MRC National Institute for Medical Research, London NW7 1AA, UK*

[2]*Division of Molecular Biosciences, Imperial College London,London SW7 2AZ, UK*

[3]*Institute for Research in Biomedicine, 6500 Bellinzona, Switzerland*

[4]*Department of Chemistry, University of Cambridge, Cambridge CB2 1EW, UK*

[5]*King's College London, London SE4, UK*

Supplementary Information

Table S1. Values of HN RDC in Phages at high salt

**Phages 500mM NaCl**		
Seq.No.	Res.	RDC
2	GLU	-3.96
3	SER	7.16
4	ILE	-0.75
5	PHE	15.01
7	GLU	15.7
8	LYS	9.89
9	GLN	5.16
11	GLY	4.57
12	SER	9.47
13	LEU	11.82
15	ALA	-5.48
17	HIS	-2.53
18	CYS	-9.32
20	ASN	3.53
21	ASN	-14.07
23	LEU	0.18
25	GLY	-9.17
27	TYR	7.80
28	PHE	-4.73
29	SER	-3.37
32	GLU	-2.20
34	SER	7.71
35	SER	-15.96
36	ILE	-9.53
38	HIS	-5.76
47	ARG	-2.73
51	GLY	2.02
53	VAL	1.50
54	THR	6.72

55	SER	-7.96
57	ASP	4.70
60	THR	4.13
62	LEU	-0.06
64	GLN	1.99
66	SER	-4.99
67	GLY	2.53
68	ASN	-6.89
71	ASP	-2.99
72	SER	6.17
79	VAL	1.88
80	ILE	-3.02
81	SER	-9.60
83	ALA	-0.66
84	LEU	-6.26
88	GLY	-5.40
90	GLU	-5.21
91	LEU	-9.81
92	ILE	-12.77
93	LEU	-6.28
94	PHE	20.41
96	SER	0.68
98	GLU	1.85
99	TYR	-2.15
100	GLN	-8.92
102	LEU	13.39
103	ARG	0.02
105	ASP	0.04
107	ILE	17.65
108	ASN	0.49
110	ARG	-12.53
111	SER	9.85
112	PHE	6.99
113	ILE	9.73
115	ASN	27.84
116	TYR	17.57
117	LYS	18.28
118	GLU	14.58
119	HIS	5.76
120	TRP	21.8
121	PHE	18.25
122	THR	29.84
123	VAL	-1.00
124	ARG	4.30
126	LEU	0.61
127	GLY	-2.56
128	LYS	-2.19
129	GLN	8.99
130	TRP	6.26
131	PHE	-2.38
132	ASN	-7.89
133	LEU	3.62
135	SER	-2.17
137	LEU	5.52
138	THR	7.05

142	LEU	0.08
143	ILE	0.40
144	SER	0.54
145	ASP	-13.86
146	THR	-4.07
148	LEU	-12.30
149	ALA	-5.22
150	LEU	-12.51
151	PHE	-9.69
154	GLN	-16.29
155	LEU	-8.46
158	GLU	-8.64
159	GLY	16.16
161	SER	24.14
162	ILE	17.00
163	PHE	14.95
164	VAL	-3.09
165	VAL	-13.15
166	LYS	-10.59
167	GLY	4.09
168	ASP	-3.41
169	LEU	8.27
171	ASP	-1.42
172	CYS	12.17
173	GLU	10.48
174	ALA	-6.73
175	ASP	3.95
176	GLN	11.01
177	LEU	-1.25
181	ILE	0.89
182	ARG	4.34

Table S2. RDC collected in phages at low ionic strength.

**Phages no salt**		
Seq.No.	Res.	RDC
2	GLU	0.54
3	SER	-0.03
4	ILE	0.15
5	PHE	0.01
7	GLU	0.57
8	LYS	1.13
9	GLN	4.22
10	GLU	-1.8
11	GLY	0.56
12	SER	0.28
13	LEU	-0.76
15	ALA	-0.59
17	HIS	-0.43
19	LEU	11.96
21	ASN	1.25

23	LEU	-0.97
25	GLY	-0.11
27	TYR	-0.52
28	PHE	0.08
34	SER	0.23
35	SER	2.74
36	ILE	-0.45
38	HIS	0.04
46	MET	-2.39
51	GLY	0.19
52	GLY	9.3
53	VAL	0.25
54	THR	-0.06
55	SER	-5.9
56	GLU	1.03
57	ASP	-2.65
59	ARG	-0.08
60	THR	0.08
62	LEU	-0.35
64	GLN	0.75
66	SER	-0.76
67	GLY	-2.14
68	ASN	-1.83
71	ASP	0.27
72	SER	1.3
79	VAL	-1.32
80	ILE	-0.31
81	SER	-0.78
83	ALA	0.05
86	VAL	-2.02
90	GLU	0.77
91	LEU	1.08
93	LEU	-0.02
96	SER	-1.48
98	GLU	1.29
99	TYR	-0.62
100	GLN	4.41
102	LEU	-0.85
105	ASP	-0.49
107	ILE	0.13
110	ARG	-0.36
111	SER	-0.19
112	PHE	0.71
113	ILE	0.2
115	ASN	1.27
116	TYR	0.96
117	LYS	0.62
118	GLU	-0.43
119	HIS	0.48
120	TRP	1.64
121	PHE	-0.64
122	THR	0.32
123	VAL	0.83
124	ARG	1
126	LEU	-0.21

128	LYS	-0.38
130	TRP	1.09
132	ASN	-0.89
133	LEU	-1.08
135	SER	8.68
136	LEU	-3.09
138	THR	-0.06
139	GLY	0.2
142	LEU	-0.06
143	ILE	-0.43
145	ASP	-0.27
146	THR	0.03
148	LEU	0.24
149	ALA	-0.14
150	LEU	0.23
151	PHE	-1.79
154	GLN	-0.27
155	LEU	-1.34
158	GLU	-0.73
159	GLY	-0.03
161	SER	0.36
162	ILE	0.44
163	PHE	0.42
164	VAL	0.08
165	VAL	0.03
166	LYS	-0.05
167	GLY	-0.06
168	ASP	-0.69
169	LEU	-0.29
171	ASP	-0.84
172	CYS	0
173	GLU	0.23
174	ALA	-0.83
175	ASP	-2.95
176	GLN	-0.5
177	LEU	0.17
181	ILE	-1.46
182	ARG	-0.08

Table S3. RDC collected in gels

**Gels**		
Seq.No.	Res.	RDC
4	ILE	8.5
7	GLU	-2.1
11	GLY	6.2
24	GLN	2.3
25	GLY	-3.7
38	HIS	-0.7
51	GLY	2.3

54	THR	5.1
60	THR	-1.4
67	GLY	10
72	SER	4.6
79	VAL	8.6
93	ALA	-0.3
94	LEU	-1.7
111	SER	-14.3
112	PHE	1
116	PHE	-1.2
118	GLU	2.2
121	PHE	4.1
123	VAL	-5.43
124	ARG	2.2
126	LEU	6.2
127	GLY	6.6
131	PHE	2.1
132	ASN	0.3
138	THR	8.1
139	LEU	7.7
142	LEU	-1
143	ILE	2.1
145	ASP	-4.3
149	ALA	-4.2
150	LEU	1.2
159	GLY	-2.4
161	SER	0.2
163	PHE	-7
164	VAL	-3.6
165	VAL	5.3
167	GLY	-4.8
169	LEU	-7

Table S4. RDC collected in doped bicelles.

**doped Bicelles**		
Seq.No.	Res.	RDC
2	GLU	-0.29
3	SER	0.17
4	ILE	-0.24
5	PHE	-5.50
8	LYS	-0.38
9	GLN	2.38
10	GLU	-0.24
11	GLY	-2.56
13	LEU	-0.79
15	ALA	0.50
17	HIS	0.03
19	LEU	-0.64
21	ASN	0.13

23	LEU	-7.32
25	GLY	0.27
27	TYR	-0.39
28	PHE	-0.94
34	SER	-0.23
35	SER	0.70
36	ILE	0.87
38	HIS	-2.29
46	MET	-2.07
51	GLY	-0.53
53	VAL	-1.21
54	THR	-2.10
55	SER	-4.83
56	GLU	2.29
59	ARG	-1.92
60	THR	0.18
64	GLN	-0.29
66	SER	-1.18
67	GLY	2.62
68	ASN	1.07
72	SER	0.63
79	VAL	0.25
80	ILE	-0.29
81	SER	-0.28
83	ALA	-0.03
86	VAL	0.62
90	GLU	0.50
91	LEU	0.04
93	LEU	0.07
96	SER	-1.16
98	GLU	1.35
99	TYR	0.07
100	GLN	-0.23
102	LEU	-0.31
105	ASP	-0.33
107	ILE	-2.14
110	ARG	-1.22
111	SER	-0.15
112	PHE	-0.26
113	ILE	-0.99
115	ASN	0.38
116	TYR	0.70
117	LYS	0.42
118	GLU	0.84
119	HIS	3.12
120	TRP	0.02
121	PHE	-0.67
122	THR	-0.09
123	VAL	0.95
124	ARG	0.23
126	LEU	0.15
128	LYS	-0.20
130	TRP	0.01
132	ASN	-0.26
135	SER	5.53

136	LEU	-1.04
138	THR	-0.17
139	GLY	0.18
142	LEU	-0.02
143	ILE	0.46
145	ASP	0.06
146	THR	-0.50
148	LEU	0.28
149	ALA	0.10
150	LEU	0.21
151	PHE	0.22
154	GLN	-0.41
155	LEU	-0.63
158	GLU	-1.06
159	GLY	-0.45
161	SER	-0.08
162	ILE	-0.23
163	PHE	-0.4
164	VAL	-0.07
165	VAL	0.11
166	LYS	-0.17
167	GLY	-0.27
168	ASP	-0.01
169	LEU	-1.50
171	ASP	0.14
172	CYS	0.15
173	GLU	0.27
175	ASP	3.95
176	GLN	-5.28
177	LEU	-3.33
181	ILE	0.17
182	ARG	-0.08
